# Changes in Cerebrospinal Fluid Balance of TNF and TNF Receptors in Naïve Multiple Sclerosis Patients: Early Involvement in Compartmentalised Intrathecal Inflammation

**DOI:** 10.3390/cells10071712

**Published:** 2021-07-06

**Authors:** Roberta Magliozzi, Francesco Pezzini, Mairi Pucci, Stefania Rossi, Francesco Facchiano, Damiano Marastoni, Martina Montagnana, Giuseppe Lippi, Richard Reynolds, Massimiliano Calabrese

**Affiliations:** 1 Department of Neuroscience, Biomedicine and Movement Science, University of Verona, 37134 Verona, Italy; francesco.pezzini@univr.it (F.P.); mairi.pucci_01@studenti.univr.it (M.P.); dam_87@hotmail.it (D.M.); martina.montagnana@univr.it (M.M.); giuseppe.lippi@univr.it (G.L.); calabresem@hotmail.it (M.C.); 2 Department of Brain Sciences, Department of Medicine, Imperial College London, London W12 0NN, UK; r.reynolds@imperial.ac.uk; 3 Department of Oncology and Molecular Medicine, Higher Institute of Health Care, 00161 Rome, Italy; stefania.rossi@iss.it (S.R.); francesco.facchiano@iss.it (F.F.); 4 Centre for Molecular Neuropathology, Lee Kong Chian School of Medicine, Singapore 308232, Singapore

**Keywords:** multiple sclerosis, TNF, cerebrospinal fluid, pathway analysis, inflammation

## Abstract

An imbalance of TNF signalling in the inflammatory milieu generated by meningeal immune cell infiltrates in the subarachnoid space in multiple sclerosis (MS), and its animal model may lead to increased cortical pathology. In order to explore whether this feature may be present from the early stages of MS and may be associated with the clinical outcome, the protein levels of TNF, sTNF-R1 and sTNF-R2 were assayed in CSF collected from 122 treatment-naïve MS patients and 36 subjects with other neurological conditions at diagnosis. Potential correlations with other CSF cytokines/chemokines and with clinical and imaging parameters at diagnosis (T0) and after 2 years of follow-up (T24) were evaluated. Significantly increased levels of TNF (fold change: 7.739; *p* < 0.001), sTNF-R1 (fold change: 1.693; *p* < 0.001) and sTNF-R2 (fold change: 2.189; *p* < 0.001) were detected in CSF of MS patients compared to the control group at T0. Increased TNF levels in CSF were significantly (*p* < 0.01) associated with increased EDSS change (r = 0.43), relapses (r = 0.48) and the appearance of white matter lesions (r = 0.49). CSF levels of TNFR1 were associated with cortical lesion volume (r = 0.41) at T0, as well as with new cortical lesions (r = 0.56), whilst no correlation could be found between TNFR2 levels in CSF and clinical or MRI features. Combined correlation and pathway analysis (ingenuity) of the CSF protein pattern associated with TNF expression (encompassing elevated levels of BAFF, IFN-γ, IL-1β, IL-10, IL-8, IL-16, CCL21, haptoglobin and fibrinogen) showed a particular relationship to the interaction between innate and adaptive immune response. The CSF sTNF-R1-associated pattern (encompassing high levels of CXCL13, TWEAK, LIGHT, IL-35, osteopontin, pentraxin-3, sCD163 and chitinase-3-L1) was mainly related to altered T cell and B cell signalling. Finally, the CSF TNFR2-associated pattern (encompassing high CSF levels of IFN-β, IFN-λ2, sIL-6Rα) was linked to Th cell differentiation and regulatory cytokine signalling. In conclusion, dysregulation of TNF and TNF-R1/2 pathways associates with specific clinical/MRI profiles and can be identified at a very early stage in MS patients, at the time of diagnosis, contributing to the prediction of the disease outcome.

## 1. Introduction

TNF is one of the main inflammatory mediators involved in several pathological conditions, including chronic inflammatory diseases such as multiple sclerosis (MS). TNF can be produced by several cell types, including immune cells (macrophages and T cells) as well as central nervous system (CNS) cells (astrocytes and microglia). It plays a pivotal role in many aspects of immune system development, immune response regulation and T cell-mediated tissue injury [1], and may have both pro-inflammatory and immune-regulatory properties, influencing the generation of the T cell repertoire, antigen-presenting cell function and apoptosis of potentially autoreactive T cells [2]. These two different effects are related to the activation of different inflammatory signalling pathways after binding to the two different receptors: TNF-R1 and TNF-R2. TNFRs are membrane receptors that can also be converted into soluble forms (sTNF-R1 and sTNF-R2) through the activity of TACE enzymes. Both soluble receptors can interact with either sTNF or mTNF, the two conformations of TNF. By binding preferably to TNF-R1, soluble TNF (sTNF) can mediate apoptosis, necroptosis or pro-inflammatory signalling, while membrane TNF (mTNF) can bind to both TNF-R1 and TNF-R2 to activate pathways involved in the resolution of inflammation and tissue regeneration [3]. Under inflammatory conditions, TNF-R1 expression can be induced in several cell types and activated by both mTNF and sTNF, while TNF-R2 is restricted to endothelial cells, various immune cells and certain CNS cells and can be effectively stimulated by mTNF [4,5].

TNF has been detected both in the cerebrospinal fluid (CSF) of MS patients in correlation with disease activity [6] and in MS white matter plaques, where it is suggested to enhance inflammation and oligodendrocyte cell death [7].

We have recently shown that the balance of the TNF signalling in post-mortem MS subpial cortical grey matter (GM) strongly correlates with the degree of meningeal inflammation and is crucial in the evolution of the neurodegenerative pathology [8]. These data were further validated by recent experimental models showing that persistent intrathecal expression of TNF, in the presence of IFNγ, is a potent inducer of meningeal inflammation and can lead to necroptotic neuronal death and subpial demyelination, and thus may contribute to more severe clinical progression in MS [9,10].

Increased levels of TNF in the CSF of MS patients could be due to the pro-inflammatory activity of resident activated glial cells, as well as the compartmentalised immune activity within perivascular and meningeal infiltrates. In particular, we have previously demonstrated in post-mortem MS cases with more extensive cortical pathology and more rapid and severe disease progression that inflammatory cells infiltrating the leptomeninges may represent a key source of TNF that is released in the CSF and may then mediate tissue injury in the underlying cortical and deep grey matter [6,11]). In addition, a high level of CSF TNF at the time of diagnosis was found to be associated with increased risk of Gad+ WML, new WML, the number of relapses and worsening EDSS after 4 years [12].

Here we aimed to perform a comprehensive evaluation of the clinical and MRI correlates of the CSF presence of TNF and its soluble receptors, TNF-R1 and TNF-R2, in treatment-naïve MS patients and the potential association with other inflammatory mediators.

## 2. Materials and Methods

### 2.1. MS Patient Cohorts 

One hundred and twenty-two consecutive treatment naïve relapsing-remitting MS patients arriving at the MS Centre of Verona University Hospital (Italy) were enrolled at diagnosis between September 2014 and February 2015. All the MS patients had a diagnosis of MS [13] (Polman 2011) and underwent neurological evaluation, 3T-MRI and CSF examination at the time of diagnosis (T0). Each patient was then followed yearly from a clinical and imaging point of view for 2 years (T24). The local Ethics Committee approved the study. Informed consent was obtained from the patients (Protocol number 66418, 25 November 2019). Biological material was obtained from voluntary donors in compliance with the Legislative Decree 196/2003 “Personal Data Protection Code”. Clinical and demographic details of the examined MS population are reported in Table 1.

### 2.2. Control Population

Thirty-six age- and sex-matched patients affected by other neurological diseases, who underwent neurological evaluation and CSF examination at the time of the diagnosis, were included in the study. This group included 21 individuals with non-inflammatory neurological diseases (NIND) (1 idiopathic tremor, 2 migraine, 2 amyloid angiopathy, 2 fibromyalgia, 4 ischemic stroke, 1 spondylotic myelopathy, 2 amyotrophic lateral sclerosis, 1 olivopontocerebellar atrophy, 1 idiopathic spastic paraparesis, 1 idiopathic ataxia, 1 myopathy, 1 endocranial hypertension, 2 peripheral neuropathy), and 15 subjects with other inflammatory neurological diseases (OIND) (1 infective myelopathy, 2 CNS lymphoma, 2 intracranial abscess, 1 peripheral neuropathy, 2 Behcet’s disease, 3 neuromyelitis optica spectrum disorder, 3 autoimmune encephalitis, 1 aseptic meningitis). The Ethics Committee of the University of Verona approved the study, and informed consent was obtained from all participants.

### 2.3. MRI Acquisition Protocol and Analysis

For each patient, an MRI was performed at least 2 months after the last relapse. MRI sequences were acquired using a Philips Achieva 3T MR Scanner. The following image sets were acquired: 3D T1 weighted; 3D double-inversion recovery (DIR); 3D Fluid Attenuated Inversion Recovery (FLAIR); 3D Echo Planar Susceptibility Weighted Imaging (EPI-SWI). Optimised parameters for each sequence were set as previously published [14] (Calabrese and Castellaro, 2017).

#### 2.3.1. WM and CL Lesion Detection and Lesion Load Assessment

White matter lesions were assessed by a consensus of experienced observers. Attention was paid to manually check the WM lesion map to identify and segment WM lesions, thus obtaining a T2-hyperintense WM lesion volume (T2WMLV). A similar consensus procedure was followed for the detection of CLs on DIR images, following recommendations for scoring of CLs in patients with MS [15]. Moreover, CLs were sub-divided into leukocortical and intracortical based on visual assessment and the cortical lesion volume was obtained as previously described [16].

#### 2.3.2. Cortical Thickness Evaluation and T1 Parcellation

The estimation of the cortical thickness was performed using the 3D T1 sequence and the automated, volume-based advanced normalisation tools (ANTs) and, in particular, the ANTs cortical thickness pipeline. The mean cortical thickness in the whole GM was calculated from the segmentation obtained with the cortical thickness pipeline. The segmentation of T2 hyperintensity was used as a mask for the lesion filling of 3D T1 images. The lesion filling was performed with the lesion filling routine included in the lesion segmentation tool [17]. The multi-atlas label fusion (MALF) technique implemented in ANTs [18] was used to parcellate the sub-cortical GM (Thalamus, Putamen, Caudate and Pallidum). The joint label fusion technique was then used to produce the final parcellation labelling. 

### 2.4. Immunoassay CSF Protein Analysis 

CSF samples were obtained at least 2 months after the last relapse and within one week of the MRI (ethical approval n° 35315), according to consensus guidelines for CSF and blood biobanking [19]. After centrifugation, the supernatant and the cell pellet were stored separately at −80 °C until use. The IgG index and presence/absence of oligoclonal bands (OCB) for each MS patient are reported in Table 1. The CSF analysis was optimised and performed by two independent investigators (R.M. and S.R.), blinded with respect to the clinical and MRI features. The levels of 69 inflammatory mediators (Supplementary Table S1) were assessed using a combination of immune-assay multiplex techniques based on the Luminex technology (40- and 37-Plex, Bio-Plex X200 System equipped with a magnetic workstation, BioRad, Hercules, CA, USA) as previously optimised [6] (Magliozzi et al., Ann Neur 2018). All samples were run in duplicate, and a number of the molecules were analysed using different immune-assay platforms in order to verify the reproducibility and consistency of the results. The CSF level of each protein detected during the analysis was normalised to the protein concentration of each CSF sample as determined by the Bradford procedure (Supplementary Table S1). When comparing the two groups of 21 NIND and 15 OIND controls, no differences were found in the presence and levels of the examined molecules; therefore, the two groups were included as a single control group (Supplementary Table S1). The levels of neurofilament light chain (NF-L) were measured using the human NF-light enzyme-linked immunosorbent assays (ELISA) kit (MyBioSource, San Diego, CA, USA) and ELISA kit (#MBS135523, MyBiosource) for fibrinogen total antigen, using VICTORTM X3 2030 Multilabel Plate Reader (Perkin Elmer, Walluf, Germany) according to the procedures previously optimised [20]. 

### 2.5. Bioinformatic Investigation and Pathway Analysis

The CSF molecular profiles were scrutinised with ingenuity pathway analysis (IPA, Qiagen, December 2020 Winter Release), which encompassed a manually curated knowledge database of the relationship among molecules, signalling pathways and functional annotations, based on the literature evidence. A core (functional) analysis was performed on the clusters of molecules associated with TNF, sTNF-R1 and sTNF-R2, as well as on the CSF profile of MS-High patients (as compared to MS-Low patients). IPA canonical pathways (CPs) related to cellular immune response, humoral immune response, cytokine signalling and nervous system signalling, as well as disease and functions categories, were interrogated to determine the most relevant functional signatures and to produce heatmaps and networks. Fisher’s exact test followed by Benjamini and Hochberg (B-H) multiple testing correction was used to calculate whether the likelihood of the correlation between molecules of the CSF profile and a specific annotation (either canonical pathway or disease biofunction) was due to random chance. IPA z-score was also utilised to estimate the activation state of a given functional annotation (positive z-score predicts an activation; negative z-score predicts an inhibition) [21]. Thresholds were set to *p*-value < 0.05 and |z-score| > 0.5. In some cases, *p*-value was reported as the −log10; in this case, a *p*-value < 0.05 corresponds to −log10(*p*-value) > 1.3. 

### 2.6. Statistics

Non-parametric Mann–Whitney U tests were used to test differences in MRI, EDSS and proteomic data between MS and control groups, as well as between MS-Low and MS-High groups. Spearman correlation coefficients were calculated to analyse the strength of correlation between clinical, MRI and CSF proteomic data, while Pearson correlation was used to analyse CSF inter-molecular correlations. A false discovery rate (FDR) with a significance level of 0.05 was adopted to correct for the multiple testing problem. Statistical analysis was performed by using GraphPad PRISM-GraphPad Software, version 7 (GraphPad Software 2365 Northside Dr. Suite 560 San Diego, CA 92108, USA).

## 3. Results

### 3.1. Differential TNF/TNFRs Protein Expression at Time of Diagnosis

**Figure 1:**
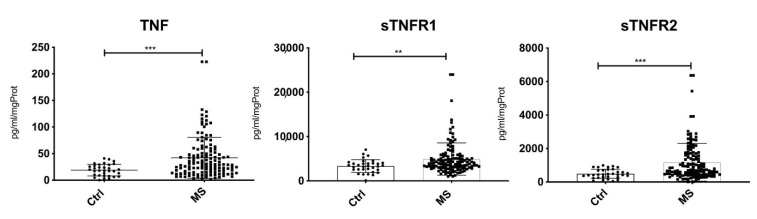
Significant increased levels of TNF (*** *p* < 0.001), sTNFR1 (** *p* < 0.01) and sTNFR2 (*** *p* < 0.001) were detected in the CSF of MS patients compared to the control group at time of diagnosis.

**Figure 2:**
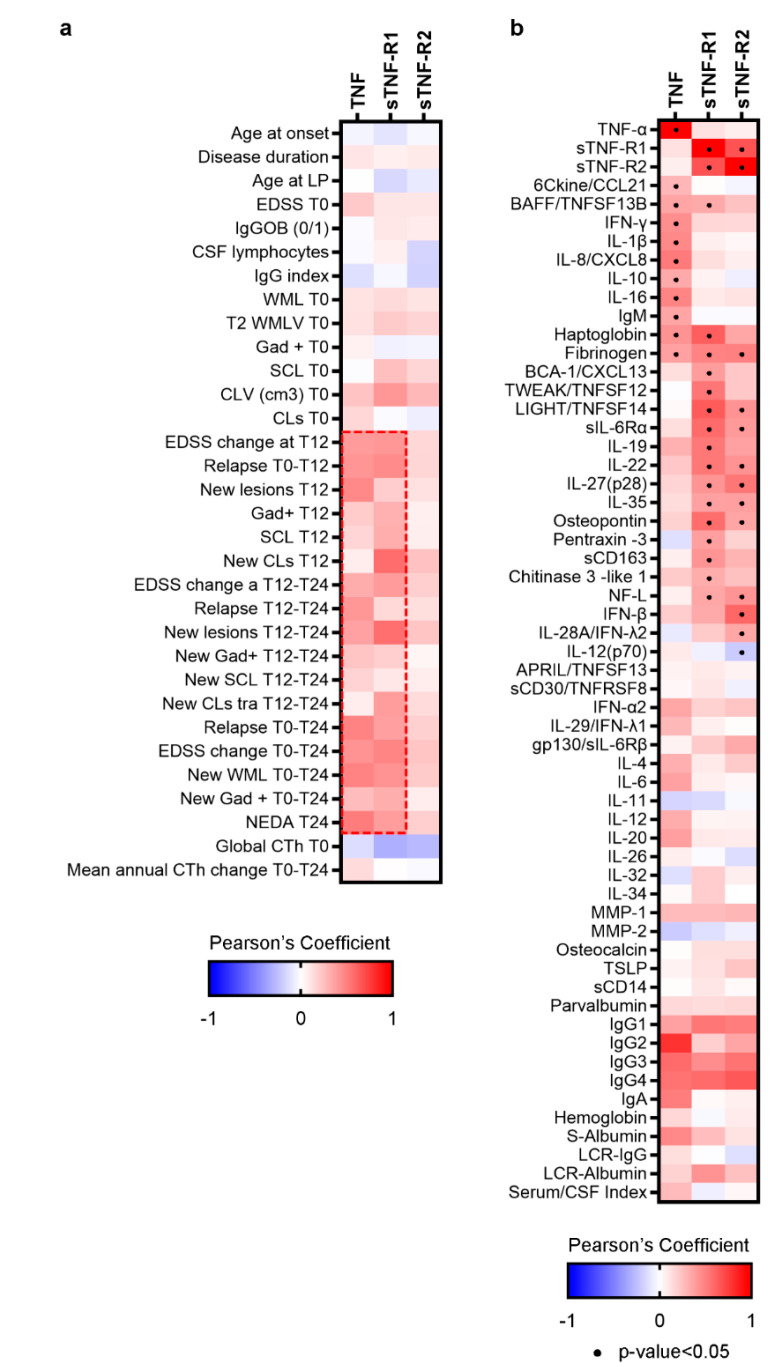
(**a**)**.** Matrix indicating significant correlations (Spearman) between the examined clinical and MRI parameters and the CSF levels of TNF and sTNFRs. Blue colour shows negative correlation, red colour shows positive correlation; strong colour tonality identifies a strong correlation. (**b**)**.** Matrix indicating significant correlations (Pearson) between the CSF levels of TNF and sTNFRs and the other examined molecules. Blue colour shows negative correlation, red colour shows positive correlation; strong colour tonality identifies a strong correlation.

Increased levels of TNF (fold change: 7.739; *p* < 0.001), sTNF-R1 (fold change: 1.693; *p* < 0.001) and sTNF-R2 (fold change: 2.189; *p* < 0.001) were detected in CSF of MS patients compared to the control group at T0 (Figure 1). The increased TNF levels were associated (*p* < 0.01) with increased EDSS change (r = 0.43), relapse rate (r = 0.48) and new white matter lesions (r = 0.49) at T24. Elevated CSF levels of TNF-R1 were associated with higher cortical lesion volumes (r = 0.41) at T0, as well as with new cortical lesions (r = 0.56), whilst no correlation could be found between TNF-R2 levels in CSF and clinical or MRI features (Figure 2a). 

### 3.2. A Specific Pattern Related to Chronic Inflammation, Acute Phase Response, Dendritic Cells and Neuroinflammation Is Associated with CSF Over-Expression of TNF and sTNFRs

By examining the inflammatory pattern associated with TNF/TNFRs CSF expression, increased levels of TNF were found to be significantly (*p* < 0.05) associated with elevated levels of BAFF, IFN-γ, IL-1β, IL-10, IL-8, IL-16, CCL21, haptoglobin and fibrinogen (Figure 2b). Increased levels of TNF-R1 were significantly (*p* < 0.05) associated with high levels of CXCL13, TWEAK, LIGHT, IL-35, osteopontin, pentraxin-3, sCD163 and chitinase-3-L1 (Figure 2b). Finally, increased levels of TNFR2 were significantly (*p* < 0.05) associated with high CSF levels of IFN-β, IFN-λ2 and sIL-6Rα (Figure 2b).

**Figure 3:**
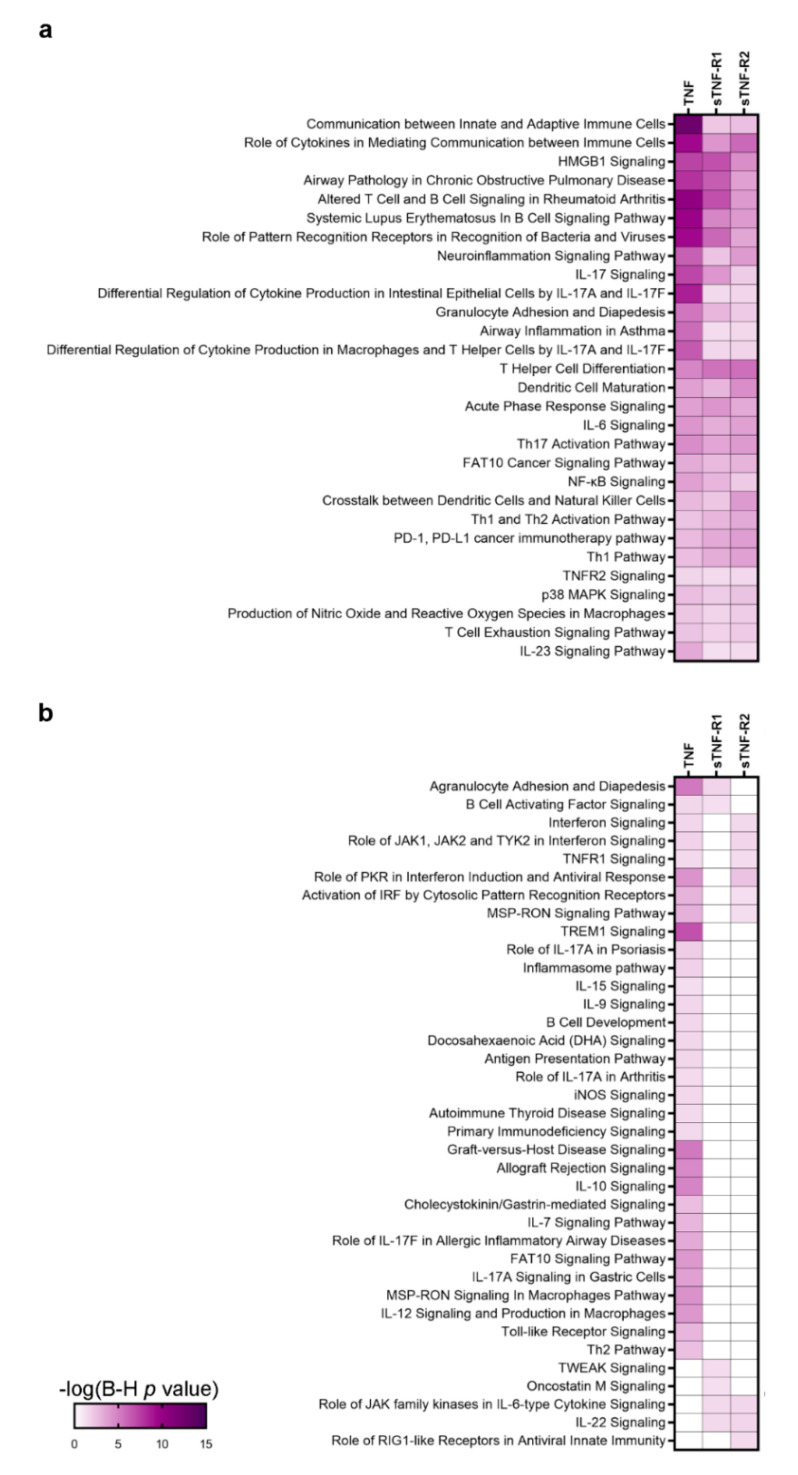
IPA canonical pathway analysis of TNF, sTNF-R1 and sTNF-R2 molecular patterns. (**a**) Heatmap of CPs shared among the three profiles pinpointed, in general, a stronger association between inflammatory pathways and TNF profile, as compared to sTNF-R1 and sTNF-R2 profiles. Communication between innate and adaptive immune cells, altered T cell and B cell signalling in rheumatoid arthritis and systemic lupus erythematosus in B cell signalling pathway showed lower *p*-value in the TNF molecular pattern. HMGB1 signalling, and IL-17 signalling were annotated to a similar extent in TNF and sTNF-R1 profiles. The role of cytokines in mediating communication between immune cells, T helper cell differentiation and dendritic cell maturation were the most significant CPs which characterised the sTNF-R2 profile. Other CPs (including acute phase response signalling, IL-6 signalling, Th17 activation pathway, Th1 and Th2 activation pathway, PD-1, PD-L1 cancer immunotherapy pathway) were shared among the three molecular patterns without remarkable difference in terms of *p*-value. (**b**) Heatmap of CPs associated with at least one or two molecular profiles indicated that TNF profile was characterised by the higher number of CPs when compared to the other two molecular patterns. Among them, CPs related to TREM-1 signalling, B cells development and IL-10 signalling were significantly annotated in the TNF profile only. For a comprehensive list of molecules enclosed in each CPs see Supplementary Table S2.

By performing pathway analysis of the CSF inflammatory patterns associated with TNF or TNF-R1/2 expression, we found that the TNF-associated profile encompassed the highest number of canonical pathways (n = 66) compared to the sTNF-R1 and sTNF-R2 patterns (n = 35 and n = 38, respectively; Figure 3a,b). Twenty-nine pathways were shared among the three patterns, but the TNF-associated profile showed lower *p*-values in general (i.e., higher −log10(*p*-value)) compared to the other two profiles, which were fairly similar (Figure 3a). Communication between innate and adaptive immune cells, systemic lupus erythematosus in B cell signalling pathway and altered T cell and B cell signalling in rheumatoid arthritis were more significantly represented in the TNF profile compared to sTNF-R1 and sTNF-R2 (darker purple square in Figure 3a). HMGB1 signalling and IL-17 signalling were shared with a similar *p*-value between TNF and sTNF-R1 profiles. Other pathways related to TREM-1 signalling, B cell functions and IL-10 were significantly represented in the TNF profile only (Figure 3b). The sTNF-R2 profile was mainly associated with the role of cytokines in mediating communication between immune cells and T helper cell differentiation, whereas other associated pathways showed less significant *p*-values compared to the other two profiles. Finally, neuroinflammation signalling pathway, dendritic cell maturation, acute phase response signalling and IL-6 signalling were shared with similar *p*-values between the TNF and TNF-R1/2 patterns.

### 3.3. Highest CSF Levels of TNF, sTNFR1 and sTNFR2 Were Present in MS Patients with Increased Cortical Lesion Loads at the Time of Diagnosis

**Figure 4:**
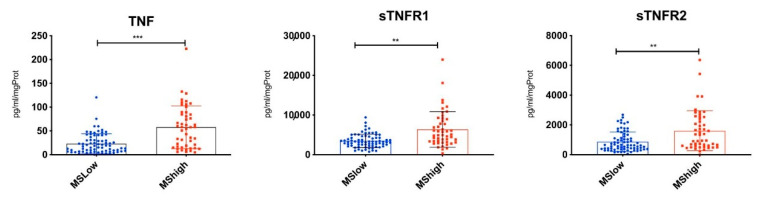
CSF levels of TNF and sTNFRs in MS patients stratified according to the high (MS-High) or low (MS-Low) cortical lesion load. Significant increased levels of TNF (*** *p* < 0.001), sTNFR1 (** *p* < 0.01) and sTNFR2 (** *p* < 0.01) were detected in CSF of MS-High compared to MS-Low patients at time of diagnosis.

When we stratified MS patients according to the cortical lesion load, we found significantly higher levels of TNF (*p* < 0.001), sTNF-R1 (*p* < 0.001) and sTNF-R2 (*p* < 0.01) in MS-High patients (with high cortical lesion loads) with respect to MS-Low ones (with low cortical lesion loads) (Figure 4). By analysing the CSF molecular profile associated with the TNF/TNFRs differential expression in more detail, in the MS-High compared to the MS-Low patients, 16 molecules were found to be differentially expressed in MS-High patients together with TNF and its TNFRs, including 14 up-regulated and 2 down-regulated (Table 2). 

**Figure 5:**
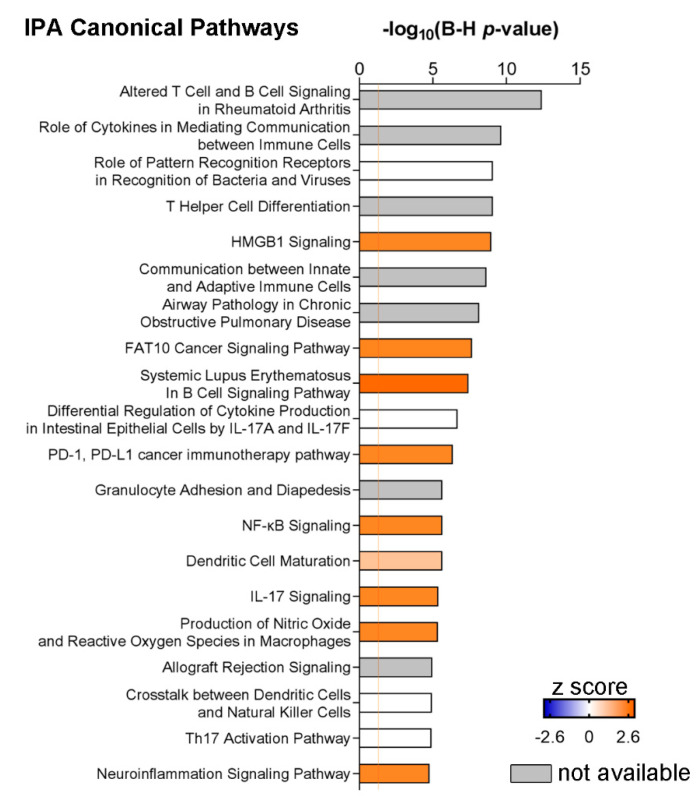
Most significant IPA canonical pathways recognised in the molecular profile of MS-High patients as compared to MS-Low patients. *HMGB1* signalling and systemic lupus erythematosus in B cell signalling pathway exhibited a positive z-score, predicting an activation state; other CPs related to T and B cells (altered T cell and B cell signalling in rheumatoid arthritis, role of cytokines in mediating communication between immune cells, T helper cell differentiation and communication between innate and adaptive immune cells) were significantly annotated, without an activation score. Furthermore, other CPs with a significant z-score were PD-1 PD1-L cancer immunotherapy pathway, IL-17 signalling and neuroinflammation signalling pathway. For the complete list of CPs of MS-High profile see Supplementary Table S3.

Following IPA core analysis of this CSF molecular profile, high-ranked pathways were found to be related to altered T and B cell processes and to innate and adaptive immunity, including: Altered T cell and B cell signalling in rheumatoid arthritis; role of cytokines in mediating communication between immune cells; T helper cell differentiation; and communication between innate and adaptive immune cells (−log10(*p*-value) = 10.60, 7.64, 7.37 and 6.91, respectively; Figure 5). Interestingly, systemic lupus erythematosus in the B cell signalling pathway was predicted to be activated (−log(*p*-value) = 6.46, z-score = 2.00). Other highly significant pathways with a positive z-score were related to dendritic cell function and neuroinflammation, namely dendritic cell maturation (−log(*p*-value) = 4.37, z-score = 1.00) and neuroinflammation signalling pathway (−log(*p*-value) = 3.71, z-score = 2.00).

### 3.4. CSF Expression of TNF, sTNFR1 and sTNFR2 May Represent Surrogate Markers of Meningeal Inflammation

**Figure 6:**
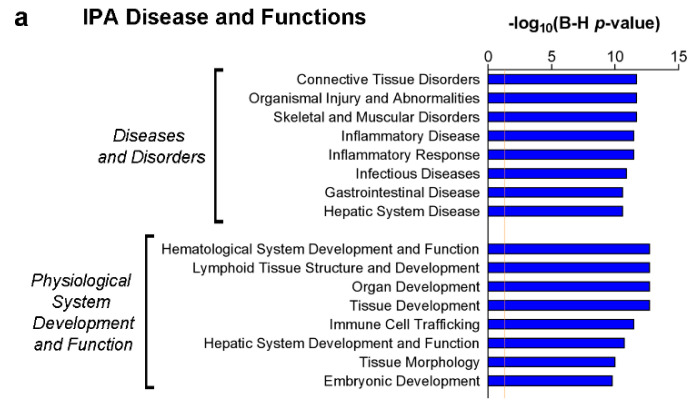

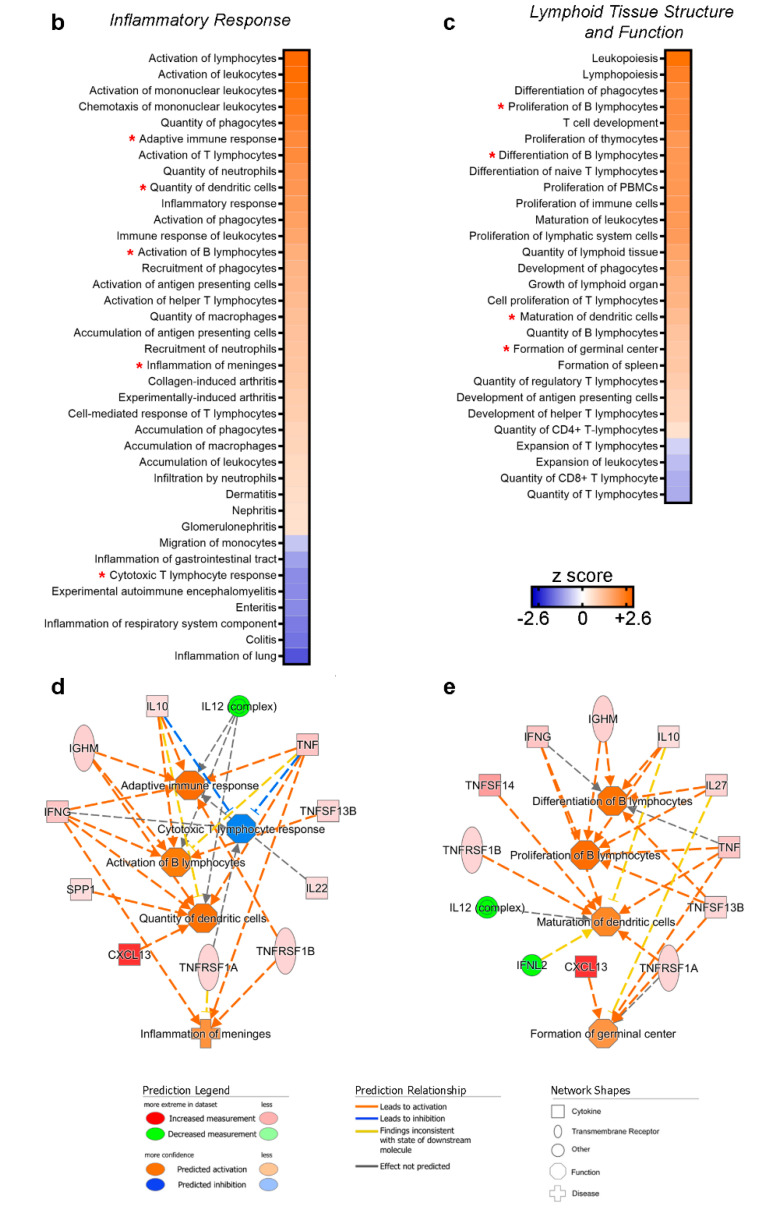
(**a**) Inquiry of disease and functions annotations revealed many categories with highly significant B-H corrected *p*-values related to inflammation (inflammatory response and disease, immunological disease) and immune cells processes (lymphoid tissue structures and development, immune cell trafficking). See Supplementary Table S4 for the complete analysis of disease and function categories. (**b**,**c**) Heatmaps of functional annotations encompassed in inflammatory response and lymphoid tissue structure and development from (**a**). All these functions showed a |z-score| > 0.5; annotations marked by red asterisk were further utilised to generate networks in panel (**d**,**e**). For the complete list of the annotations enclosed in the two categories see Supplementary Tables S5 and S6. (**d**,**e**) Networks from selected annotations of inflammatory response (**d**) and lymphoid tissue structures and development categories (**e**) pinpointed an involvement of adaptive immunity and B cell response; conversely, annotations related to T cell response were associated with negative z-score, predicting an inhibition state. Notably, inflammation of meninges was also found to be activated: this prediction was supported by the increased expression of IFN-γ, TNFRS1B/TNF-R2 and TNF (orange arrows), whereas TNFRS1A/TNF-R1 showed an inconsistent relationship (yellow arrow). Furthermore, the activation state of formation of germinal centre was supported by the increased expression of CXCL13, BAFF/TNFRSF13B and TNF (orange arrows), whereas the upregulation of IL-27 was inconsistent with the prediction (yellow arrow); the effect of the relationship with TNFRSF1A/TNF-R1 could not be predicted (grey arrow).

According to the IPA Disease and Functions analysis, the most significant categories associated with the CSF profile of MS-High patients were related to inflammatory disorders as well as to immune cell processes (bars in Figure 6a). Inflammatory response and lymphoid tissue structure and development (−log(*p*-value) = 11.51 and 12.77, respectively) were further analysed to unravel functional annotations showing a meaningful z-score. The inflammatory response encompassed many predicted activated functions related to the adaptive immune response (z-score = 2.21), as well as to dendritic cells (quantity of dendritic cells z-score = 1.97), antigen-presenting cells (accumulation and activation of antigen-presenting cells, z-score = 1.17 and 1.37) and B cells (activation of B lymphocytes z-score = 1.54; Figure 6b). Intriguingly, inflammation of meninges was also predicted to be activated in the MS-High profile (z-score = 1.11). Processes related to T lymphocytes were also present, including cytotoxic T lymphocyte response, which was predicted to be inhibited (z-score = −1.372). The investigation of lymphoid tissue structure and development category confirmed the involvement of B lymphocytes and dendritic cells (proliferation, maturation and differentiation of B lymphocytes, z-score = 2.17, 1.98 and 1.96; maturation of dendritic cells, z-score = 1.28; Figure 6c). In addition, formation of germinal centre was also significantly annotated and activated (z-score = 1.07). Some of the functional annotations related to T lymphocytes showed opposing trends, as indicated by both positive and negative z-scores (differentiation of naive T lymphocytes, z-score = 1.96; quantity of regulatory T lymphocytes, z-score = 0.95; quantity of CD8+ T lymphocyte, z-score = −0.98; quantity of T lymphocytes, z-score = −1.01). Networks of molecules in the CSF profile of MS-High patients, which contributed to the z-score of some functional annotations described above, are depicted in Figure 6d,e.

## 4. Discussion

Sustained TNF expression has a key role in the target organ pathology of several chronic inflammatory diseases, such as multiple sclerosis. Here, we have demonstrated that the overexpression of TNF and sTNFRs in the CSF of early diagnosed MS patients is associated with a particular clinical/MRI profile and a concomitant specific CSF molecular pattern. In particular, CSF levels of TNF and sTNF-R1, but not of sTNF-R2, at the time of diagnosis correlated with evidence of disease activity after 2 years, as shown by the number of relapses, new white and grey matter lesions and increased EDSS score. These data support the experimental evidence that TNF mediates pro-inflammatory events, in particular by activating TNF-R1 signalling and suggest that selective TNF/TNF-R1 inhibition rather than complete TNF blockade may reduce the pro-inflammatory effects of TNF, thus preserving relevant neuroprotective and repair signals that occur via TNF-R2 signalling [22,23,24,25]. 

The correlation found between signs of clinical disease activity in MS patients at the time of diagnosis, such as increased EDSS change, an elevated number of relapses and new white matter and cortical lesions, and CSF levels of TNF and TNF-R1, demonstrates that the CSF molecular signatures of the disease phenotype can already be identified at the earliest stages of MS [12,26,27]. In particular, CSF TNF may represent one of the best biomarkers of cortical pathology, at least in a subgroup of MS patients [6,28].

In addition, pathway analysis suggested that an imbalance in TNF/TNFRs expression in the CSF may represent a surrogate marker of meningeal inflammation and, possibly, of germinal centre formation. This result strongly supports our previous finding that the change in the balance of TNF-R1 versus TNF-R2 mediated signalling in post-mortem MS cases at the gene expression level is related to the presence of meningeal inflammation organised in tertiary lymphoid-like structures, to diffuse GM pathology and to more rapid and severe disease progression [8]. Therefore, it can be confirmed that meningeal inflammation almost certainly plays a crucial role in MS pathology since the initial stages of the disease. CSF inflammatory biomarkers, as well as imaging methodologies detecting GM lesions, may represent the only currently available tools to identify MS patients that already have a meningeal inflammation-driven phenotype at the time of diagnosis. In particular, CSF levels of TNF and TNFRs may represent one of the best correlations of intrathecal inflammation that can be examined at the time of diagnosis and possibly during the disease follow-up. 

The advanced pathway analysis revealed that different cellular and humoral response pathways were strongly associated with the TNF profile, with a partial overlap with sTNF-R1 and sTNF-R2, particularly in a subgroup of MS patients characterised by the highest level of cortical demyelination and CSF inflammation, strongly supporting a strict correlation between TNF and B cell activity in MS. However, while TNF is well known to play a key role in developmental lymphoid organogenesis and physiological B cell functions, it remains less clear whether an aberrant activation of some B cell functions or defects in regulatory B cell activity may be associated with the deregulation of TNF signalling pathways. By releasing IL-10, TGF-β, IgM and IL-35, as well as by expressing FasL, and other costimulatory factors, such as CD40, CD80/86 and PDL1, B-cells have been demonstrated to have a key role in regulating TNF expression/production by CD4 T cells and monocytes/macrophages [29]. At the same time, the strong correlation between TNF pathways and B cell activity in the CSF highlights the key role in chronic inflammatory diseases, such as MS, of the interaction between innate immune cell activity (microglia/macrophages) and adaptive B cell responses. Several molecules produced by B cells, such as IL-10 and IL-35, as well as an increased function of B cells as antigen-presenting cells (APCs), were associated with higher activation of macrophages and pro-inflammatory T cells in MS and other autoimmune diseases [30]. Therefore, it is important to better clarify the MS-specific TNF-mediated molecular mechanisms regulating B cell immunity in MS.

TNF has been proposed as a principal pro-inflammatory mediator in MS pathogenesis and its levels associated with disease progression, but, surprisingly, the systemic blockade of TNF in some MS patients led to immune activation and increased disease activity, as demonstrated by two clinical trials involving the use of antibody-based TNF antagonists in patients with demyelinating disease [31,32]. The first tested infliximab, a humanised mouse anti-TNF monoclonal antibody on two patients with RRMS, which provoked an increase in disease activity at MRI. Lenercept, a recombinant soluble TNF-R1 fusion protein, was tested in the second trial on 168 patients and caused a deterioration of the clinical course in some patients compared to controls. These results were likely to have been due to the non-selective TNF inhibition, as both effectively block sTNF and mTNF, which can have opposing effects. The use of selective inhibition of TNF-R2 with a soluble TNF-R2 fusion protein (etanercept) has also been associated with the manifestation of MS-like demyelinating lesions due to the blockade of both sTNF and mTNF, confirming the idea that mTNF probably has a dominant role in the regenerative process and resolution of inflammation [33]. It has been recently suggested that the protective function in inflamed central nervous system pathology by T regulatory cells requires the surface expression of TNF-R2, therefore explaining the potential of some adverse effects of anti-TNF therapy in patients [34].

In addition, one of the possible explanations of the anti-TNF ineffectiveness could be the limited access to the CNS, which would not allow the achievement of an adequate therapeutic level of the drugs [2]. In active MS, the permeability of the blood-brain barrier (BBB) is only mildly modified, so TNF antagonists are enabled to penetrate in CNS.

Several experimental studies in mice deficient for TNF-R1 have demonstrated that it has a detrimental role as mice were entirely protected against EAE disease, whereas TNF-R2-KO mice exhibited exacerbated disease, enhanced Th1 cytokine production and enhanced CD4+ T cell infiltration in the CNS, suggesting an immunomodulatory and protective role for TNF-R2 signalling [35,36,37]. 

Many aspects of the role and function of the TNF in MS still need to be explored: the different role and balance between sTNF and mTNF (pro-inflammatory role vs. homeostatic functions), rather than the TNF-R1/TNF-R2 ratio. We need to understand if the soluble molecules (sTNF and sTNF-R1/2) are in equilibrium with the corresponding membrane bound proteins (mTNF and TNF-R1/2) and whether the increase of TNF in the CSF in MS is dependent on B clonal expansion or not. The topic is really complex and needs to be adequately investigated with new in vitro and animal model studies. One therapeutic solution could be to intervene at the level of downstream signalling rather than the ligand-receptor interaction in order to increase the specificity against the pathogenetic process. 

## 5. Conclusions

Our study confirms that TNF is significantly elevated in the CSF of MS patients at the time of diagnosis and that soluble TNF-R1 predominates over TNF-R2 even in physiological conditions (5:1 ratio) with a greater overexpression of TNF-R1 in patients compared to controls. Since there is not a single unequivocal marker of inflammation and demyelination at early stages of MS, we think that the determination of TNF and its receptor balance in the CSF could contribute, together with advanced MRI data, to identify patients with a worse prognosis who need more aggressive treatment immediately. In addition, this study suggests that there is an imbalance between TNFR1 and TNFR2 signalling in patients with MS from the earliest clinical events and that this can have an important prognostic meaning, announcing the appearance of new cortical lesions and a clinical worsening of the disease. The differential roles of TNF in MS are mediated by multiple pathways, therefore addressing a possible shift of the balance between TNF-R1 and TNF-R2 signalling in the pathological condition could be an important avenue for MS treatment, but further studies are necessary. 

## Figures and Tables

**Table 1:** Demographic, clinical and MRI details of the MS examined population.

	Mean	Std. Deviation
Age at onset	35.36	12.24
Age at LP	37.78	12.71
EDSS T0	1.83	1.06
T2 WM lesions	8.29	3.76
T2WM lesions volume	835.10	502.70
Gad + lesions T0	0.13	0.48
Spinal cord lesions T0	0.51	0.96
CLs volume (cm3) T0	215.60	297.80
CLs number T0	7.72	5.90
EDSS change a T12	0.22	0.38
Relapse T0-T12	0.26	0.48
New T2 WM lesions T12	0.79	1.36
New Gad+ lesions T12	0.29	0.61
New spinal cord lesions T12	0.14	0.38
New CLs T12	0.88	1.48
EDSS change T12-T24	0.27	0.45
Relapse T12-T24	0.20	0.43
New T2 WM lesions T12-T24	0.70	1.12
NewGad+ lesions T12-T24	0.18	0.44
New spinal cord lesions T12-T24	0.14	0.34
New CLs T12-T24	0.82	1.31
Relapse T0-T24	0.44	0.76
EDSS change T0-T24	0.45	0.70
New T2 WM lesionsT0-T24	1.69	2.37
New Gad+ lesions T0-T24	0.46	0.85

Abbreviations: EDSS: expanded disability status scale; CL: cortical lesion; T2WMLV: T2 white matter lesion volume.

**Table 2:** CSF inflammatory pattern associated with differential cortical lesion load.

Molecule	MS-High		MS-Low		Fold Change
	Mean	StDev	Mean	StDev	
TNF-α ***	57.72	25.37	23.21	20.74	2.49
sTNF-R1 **	6367.02	545.69	3465.76	1698.50	1.84
sTNF-R2 **	1601.67	357.31	866.46	652.85	1.85
BAFF/ TNFSF13B **	10,926.91	760.80	9049.70	6004.98	1.21
LIGHT/ TNFSF14 **	663.90	105.88	156.56	256.93	4.24
IFN-γ **	24.77	9.33	9.85	14.12	2.51
BCA-1/ CXCL13 ***	27.77	3.81	3.17	4.87	8.75
IL-10 **	22.19	3.22	18.04	17.12	1.23
IL-22 *	52.64	26.72	33.62	36.19	1.57
IL-27(p28) *	271.39	51.76	139.56	171.48	1.94
Osteopontin/SPP1 **	108,631.45	10,203.80	70,400.60	81,511.52	1.54
Pentraxin-3 *	529.91	72.04	282.65	414.24	1.87
sCD163 **	55,404.70	2111.60	44,373.96	35,703.43	1.24
Chitinase 3-like 1 **	52,758.42	4285.75	37,362.73	30,994.71	1.41
IgM *	707.97	61.54	346.19	338.14	2.05
Fibrinogen *	1483.38	184.83	941.78	6.64	1.58
NF-L *	2.82	0.68	1.62	0.95	1.74
IL-28A/IFN-λ2 *	243.89	67.45	350.47	91.71	0.57
IL-12(p70) *	9.00	3.87	13.81	2.53	0.62

* *p* < 0.05; ** *p* < 0.01; *** *p* < 0.001.

## Data Availability

All the data supporting reported results can be found from the authors R.M. and F.P.

## Supplementary Materials

The following are available online at https://www.mdpi.com/article/10.3390/cells10071712/s1. Table S1: Protein mean concentration (pg/mL/mgProt) of all the examined molecules in the CSF of the MShigh, Mslow and control patients; Table S2: Most Significant Canonical Pathways annotated in TNF, sTNF-R1 and sTNF-R2 molecular patterns; Table S3: IPA Canonical Pathway associated with CSF molecular profile of MS-High patients compared to MS-Low patients; Table S4: IPA Disease and Functions categories related to CSF profile of MS-High patients compared to MS-Low patients; Table S5: Functional annotations encompassed Inflammatory Response category of MS-High patients’ CSF profile; Table S6: Functional annotations encompassed Lymphoid Tissue Structure and Development category of MS-High patients’ CSF profile.

## References

[1] Rossol M., Meusch U., Pierer M., Kaltenhäuser S., Häntzschel H., Hauschildt S., Wagner U. (2007). Interaction between Transmembrane TNF and TNFR1/2 Mediates the Activation of Monocytes by Contact with T Cells. J. Immunol..

[2] Robinson W.H., Genovese M.C., Moreland L.W. (2001). Demyelinating and neurologic events reported in association with tumor necrosis factor α antagonism: By what mechanisms could tumor necrosis factor α antagonists improve rheumatoid arthritis but exacerbate multiple sclerosis?. Arthritis Rheum..

[3] Probert L. (2015). TNF and its receptors in the CNS: The essential, the desirable and the deleterious effects. Neuroscience.

[4] Dopp J.M., Sarafian T.A., Spinella F.M., Kahn M.A., Shau H., de Vellis J. (2002). Expression of the p75 TNF receptor is linked to TNF-induced NFkappaB translocation and oxyradical neutralization in glial cells. Neurochem. Res..

[5] Pegoretti V., Baron W., Laman J.D., Eisel U.L.M. (2018). Selective Modulation of TNF–TNFRs Signaling: Insights for Multiple Sclerosis Treatment. Front. Immunol..

[6] Magliozzi R., Howell O.W., Nicholas R., Cruciani C., Castellaro M., Romualdi C., Rossi S., Pitteri M., Benedetti M.D., Gajofatto A. (2018). Inflammatory intrathecal profiles and cortical damage in multiple sclerosis. Ann. Neurol..

[7] Lepennetier G., Hracsko Z., Unger M., Van Griensven M., Grummel V., Krumbholz M., Berthele A., Hemmer B., Kowarik M.C. (2019). Cytokine and immune cell profiling in the cerebrospinal fluid of patients with neuro-inflammatory diseases. J. Neuroinflammation.

[8] Magliozzi R., Howell O.W., Durrenberger P., Aricò E., James R., Cruciani C., Reeves C., Roncaroli F., Nicholas R., Reynolds R. (2019). Meningeal inflammation changes the balance of TNF signalling in cortical grey matter in multiple sclerosis. J. Neuroinflammation.

[9] James R.E., Schalks R., Browne E., Eleftheriadou I., Munoz C.P., Mazarakis N.D., Reynolds R. (2020). Persistent elevation of intrathecal pro-inflammatory cytokines leads to multiple sclerosis-like cortical demyelination and neurodegeneration. Acta Neuropathol. Commun..

[10] Picon C., Jayaraman A., James R., Beck C., Gallego P., Witte M.E., van Horssen J., Mazarakis N.D., Reynolds R. (2021). Neuron-specific activation of necroptosis signaling in multiple sclerosis cortical grey matter. Acta Neuropathol..

[11] Gardner C., Magliozzi R., Durrenberger P.F., Howell O.W., Rundle J., Reynolds R. (2013). Cortical grey matter demyelination can be induced by elevated pro-inflammatory cytokines in the subarachnoid space of MOG-immunized rats. Brain.

[12] Magliozzi R., Scalfari A., Pisani A.I., Ziccardi S., Marastoni D., Pizzini F.B., Bajrami A., Tamanti A., Guandalini M., Bonomi S. (2020). The CSF Profile Linked to Cortical Damage Predicts Multiple Sclerosis Activity. Ann. Neurol..

[13] Polman C.H., Reingold S.C., Banwell B., Clanet M., Cohen J.A., Filippi M., Fujihara K., Havrdova E., Hutchinson M., Kappos L. (2011). Diagnostic criteria for multiple sclerosis: 2010 Revisions to the McDonald criteria. Ann. Neurol..

[14] Calabrese M., Castellaro M. (2017). Cortical Gray Matter MR Imaging in Multiple Sclerosis. Neuroimaging Clin. N. Am..

[15] Geurts J.J.G.G., Roosendaal S.D., Calabrese M., Ciccarelli O., Agosta F., Chard D.T., Gass A., Huerga E., Moraal B., Pareto D. (2011). Consensus recommendations for MS cortical lesion scoring using double inversion recovery MRI. Neurology.

[16] Calabrese M., Battaglini M., Giorgio A., Atzori M., Bernardi V., Mattisi I., Gallo P., De Stefano N. (2010). Imaging distribution and frequency of cortical lesions in patients with multiple sclerosis. Neurology.

[17] Roura E., Oliver A., Cabezas M., Valverde S., Pareto D., Vilanova J.C., Ramió-Torrentà L., Rovira À., Lladó X. (2015). A toolbox for multiple sclerosis lesion segmentation. Neuroradiology.

[18] Wang H., Yushkevich P.A. (2013). Multi-atlas segmentation with joint label fusion and corrective learning—An open source implementation. Front. Neuroinform..

[19] Teunissen C.E., Petzold A., Bennett J.L., Berven F.S., Brundin L., Comabella M., Franciotta D., Frederiksen J.L., Fleming J.O., Furlan R. (2009). A consensus protocol for the standardization of cerebrospinal fluid collection and biobanking. Neurology.

[20] Magliozzi R., Hametner S., Facchiano F., Marastoni D., Rossi S., Castellaro M., Poli A., Lattanzi F., Visconti A., Nicholas R. (2019). Iron homeostasis, complement, and coagulation cascade as CSF signature of cortical lesions in early multiple sclerosis. Ann. Clin. Transl. Neurol..

[21] Pezzini F., Bianchi M., Benfatto S., Griggio F., Doccini S., Carrozzo R., Dapkunas A., Delledonne M., Santorelli F.M., Lalowski M.M. (2017). The Networks of Genes Encoding Palmitoylated Proteins in Axonal and Synaptic Compartments Are Affected in PPT1 Overexpressing Neuronal-Like Cells. Front. Mol. Neurosci..

[22] Brambilla R., Ashbaugh J.J., Magliozzi R., Dellarole A., Karmally S., Szymkowski D.E., Bethea J.R. (2011). Inhibition of soluble tumour necrosis factor is therapeutic in experimental autoimmune encephalomyelitis and promotes axon preservation and remyelination. Brain.

[23] Taoufik E., Tseveleki V., Chu S.Y., Tselios T., Karin M., Lassmann H., Szymkowski D.E., Probert L. (2011). Transmembrane tumour necrosis factor is neuroprotective and regulates experimental autoimmune encephalomyelitis via neuronal nuclear factor-κB. Brain.

[24] Williams S.K., Maier O., Fischer R., Fairless R., Hochmeister S., Stojic A., Pick L., Haar D., Musiol S., Storch M.K. (2014). Antibody-Mediated Inhibition of TNFR1 Attenuates Disease in a Mouse Model of Multiple Sclerosis. PLoS ONE.

[25] Madsen P.M., Motti D., Karmally S., Szymkowski D.E., Lambertsen K.L., Bethea J.R., Brambilla R. (2016). Oligodendroglial TNFR2 mediates membrane TNF-dependent repair in experimental autoimmune encephalomyelitis by promoting oligodendrocyte differentiation and remyelination. J. Neurosci..

[26] Rossi S., Motta C., Studer V., Barbieri F., Buttari F., Bergami A., Sancesario G., Bernardini S., De Angelis G., Martino G. (2014). Tumor necrosis factor is elevated in progressive multiple sclerosis and causes excitotoxic neurodegeneration. Mult. Scler. J..

[27] Donninelli G., Studer V., Brambilla L., Zecca C., Peluso D., Laroni A., Michelis D., Mantegazza R., Confalonieri P., Volpe E. (2021). Immune Soluble Factors in the Cerebrospinal Fluid of Progressive Multiple Sclerosis Patients Segregate Into Two Groups. Front. Immunol..

[28] Bai Z., Chen D., Wang L., Zhao Y., Liu T., Yu Y., Yan T., Cheng Y. (2019). Cerebrospinal Fluid and Blood Cytokines as Biomarkers for Multiple Sclerosis: A Systematic Review and Meta-Analysis of 226 Studies With 13,526 Multiple Sclerosis Patients. Front. Neurosci..

[29] Rincón-Arévalo H., Sanchez-Parra C.C., Castaño D., Yassin L., Vásquez G. (2016). Regulatory B Cells and Mechanisms. Int. Rev. Immunol..

[30] Shen P., Roch T., Lampropoulou V., O’Connor R.A., Stervbo U., Hilgenberg E., Ries S., Dang V.D., Jaimes Y., Daridon C. (2014). IL-35-producing B cells are critical regulators of immunity during autoimmune and infectious diseases. Nature.

[31] van Oosten B.W., Barkhof F., Truyen L., Boringa J.B., Bertelsmann F.W., von Blomberg B.M.E., Woody J.N., Hartung H.-P., Polman C.H. (1996). Increased MRI activity and immune activation in two multiple sclerosis patients treated with the monoclonal anti-tumor necrosis factor antibody cA2. Neurology.

[32] Arnason B.G.W. (1999). TNF neutralization in MS: Results of a randomized, placebo-controlled multicenter study. Neurology.

[33] Seror R., Richez C., Sordet C., Rist S., Gossec L., Direz G., Houvenagel E., Berthelot J.-M., Pagnoux C., Dernis E. (2013). Pattern of demyelination occurring during anti-TNF-α therapy: A French national survey. Rheumatology.

[34] Ronin E., Pouchy C., Khosravi M., Hilaire M., Grégoire S., Casrouge A., Kassem S., Sleurs D., Martin G.H., Chanson N. (2021). Tissue-restricted control of established central nervous system autoimmunity by TNF receptor 2–expressing Treg cells. Proc. Natl. Acad. Sci. USA.

[35] Suvannavejh G.C., Lee H.O., Padilla J., Dal Canto M.C., Barrett T.A., Miller S.D. (2000). Divergent roles for p55 and p75 tumor necrosis factor receptors in the pathogenesis of MOG35-55-induced experimental autoimmune encephalomyelitis. Cell. Immunol..

[36] Arnett H.A., Mason J., Marino M., Suzuki K., Matsushima G.K., Ting J.P.Y. (2001). TNFα promotes proliferation of oligodendrocyte progenitors and remyelination. Nat. Neurosci..

[37] Fresegna D., Bullitta S., Musella A., Rizzo F.R., De Vito F., Guadalupi L., Caioli S., Balletta S., Sanna K., Dolcetti E. (2020). Re-Examining the Role of TNF in MS Pathogenesis and Therapy. Cells.

